# Genome-Wide Analysis of the DYW Subgroup PPR Gene Family and Identification of *GmPPR4* Responses to Drought Stress

**DOI:** 10.3390/ijms20225667

**Published:** 2019-11-12

**Authors:** Hong-Gang Su, Bo Li, Xin-Yuan Song, Jian Ma, Jun Chen, Yong-Bin Zhou, Ming Chen, Dong-Hong Min, Zhao-Shi Xu, You-Zhi Ma

**Affiliations:** 1 Institute of Crop Science, Chinese Academy of Agricultural Sciences (CAAS)/National Key Facility for Crop Gene Resources and Genetic Improvement, Key Laboratory of Biology and Genetic Improvement of Triticeae Crops, Ministry of Agriculture, Beijing 100081, China; suhonggang924@126.com (H.-G.S.); libo708@126.com (B.L.); chenjun01@caas.cn (J.C.); zhouyongbin@caas.cn (Y.-B.Z.); chenming02@caas.cn (M.C.); 2 State Key Laboratory of Crop Stress Biology for Arid Areas, College of Agronomy, Northwest A&F University, Yangling 712100, China; mdh2493@126.com; 3 Agro-Biotechnology Research Institute, Jilin Academy of Agriculture Sciences, Changchun 130033, China; songxinyuan1980@163.com; 4 College of Agronomy, Jilin Agricultural University, Changchun 130118, China; majian197916@jlau.edu.cn

**Keywords:** Pentatricopeptide-repeat (PPR) proteins, genome-wide analysis, drought responses, hairy root assay, soybean

## Abstract

Pentatricopeptide-repeat (PPR) proteins were identified as a type of nucleus coding protein that is composed of multiple tandem repeats. It has been reported that *PPR* genes play an important role in RNA editing, plant growth and development, and abiotic stresses in plants. However, the functions of PPR proteins remain largely unknown in soybean. In this study, 179 DYW subgroup *PPR* genes were identified in soybean genome (*Glycine max* Wm82.a2.v1). Chromosomal location analysis indicated that DYW subgroup *PPR* genes were mapped to all 20 chromosomes. Phylogenetic relationship analysis revealed that DYW subgroup *PPR* genes were categorized into three distinct Clusters (I to III). Gene structure analysis showed that most *PPR* genes were featured by a lack of intron. Gene duplication analysis demonstrated 30 *PPR* genes (15 pairs; ~35.7%) were segmentally duplicated among Cluster I *PPR* genes. Furthermore, we validated the mRNA expression of three genes that were highly up-regulated in soybean drought- and salt-induced transcriptome database and found that the expression levels of *GmPPR4* were induced under salt and drought stresses. Under drought stress condition, *GmPPR4-*overexpressing (*GmPPR4*-OE) plants showed delayed leaf rolling; higher content of proline (Pro); and lower contents of H_2_O_2_, O_2_^−^ and malondialdehyde (MDA) compared with the empty vector (EV)-control plants. *GmPPR4*-OE plants exhibited increased transcripts of several drought-inducible genes compared with EV-control plants. Our results provided a comprehensive analysis of the DYW subgroup *PPR* genes and an insight for improving the drought tolerance in soybean.

## 1. Introduction

The pentatricopeptide repeat (PPR) proteins containing tandem 30–40 amino acid sequence motifs, constitute a large gene family in plants [1]. Typically, PPR proteins are classed into two major subfamilies (P and PLS). The P subfamily proteins contain only the canonical P motif (35 amino acid residues); the PLS subfamily proteins are confined to contain a series of PLS triplets (L, long variants of P; S, small variants of P) [2]. L motif and S motif contain 31 and 35 or 36 amino acid residues, respectively, which are considered to be variations of the P motif [2]. Most PPR proteins are highly conserved at the C-terminus and usually have three conserved domains at the C-terminus, E, E+ and DYW domains, respectively [3,4]. Based on these C-terminal domains, the PLS subfamily is divided into four subgroups, PLS subgroup, E subgroup, E+ subgroup, and DYW subgroup [2]. The PPR proteins also contain a distinctive feature that is essentially free of intron. Although members of the PPR family are widely distributed in plants and are large in number, they perform special functions [2].

So far, a considerable amount of PPR proteins have been experimentally identified from various plant species. [5]. For example, 441 and 626 PPR members have been identified in the *A. thaliana* and *Populus trichoccapa* genome, respectively [5,6,7]. A large number of studies showed that PPR proteins played roles in the growth and development of plants and the formation of organelles [8]. It can participate in the fertility restoration of cytoplasmic male sterility, post-transcriptional processing of RNA and adversity defense [2,7,9,10,11,12]. In maize, EMP12 is targeted to the mitochondria and is essential for the splicing of three *nad2* introns and seed development, and the mutant in *EMP12* caused defects complex I [13]. It was reported that the nuclear *OsNPPR1* encoded a PPR protein, which was involved in the Regulation of mitochondrial development and endosperm development. The mutant named *fgr1* exhibited a lower content of starch and delayed seedling growth [14]. Rice *PPS1* encodes a DYW motif-containing PPR protein, which is important for C-to-U RNA editing of *nad3* transcripts. Knock-down and Knock-out of *PPS1* revealed a significant decrease in the editing efficiency of C-to-U at five editing sites of *nad3* transcripts, which resulted in some pleiotropic phenotypes, such as dwarfing, developmental retardation and stunted development in vegetative stages; partial pollen was sterile at the reproductive stage [15]. A chloroplast-localized P-class PPR protein, PpPPR_21, was identified as an essential protein for the accumulation of a stable *psbl-ycf12* mRNA. The knockout *ppr21* mutants displayed decreasing protonemata growth and lower photosynthetic activity [16].

Of particular note, several studies have recently reported roles for *PPR* genes during abiotic stress [17]. For example, *PPR96* from *A. thaliana* was involved in the response to salt, ABA and oxidative stress, and altered translational levels of abscisic stress responsive genes, implying that *PPR96* may take part in the regulation of plant tolerance to abiotics [18]. PGN, an *A. thaliana* mitochondria-localized PPR protein, was reported to play a role in regulating mitochondrial reactive oxygen species balance in abiotic and biotic stress responses. Inactivation of *PGN* displayed hypersensitivity to ABA, glucose, and salinity. Loss of *PGN* resulted in enhanced accumulation of reactive oxygen species (ROS) in seedlings, and the *pgn* mutant notably elevated levels of *ABI4* and *ALTERNATIVE OXIDASE1a* [19]. In rice, *cisc(t)* encoded a PPR protein, which was necessary for chloroplast development in early stages under cold stress [20]. The *A. thaliana ABA overly sensitive 5 (abo5)* mutant showed growth retardation and accumulated increased levers of H_2_O_2_ in root tips [21]. *AtPPR40* was reported to be involved in salt, ABA and oxidative stress [22].

Previous studies have emphasized the role of *PPR* genes in the growth and development of plants, and several *PPR* genes performed potential roles against abiotic stresses. To date, little information about *PPR* genes relating to abiotic stress response mechanisms in soybean has been reported. Here, a comprehensive genome-wide analysis of the DYW subgroup PPR gene family has been accomplished in soybean. We explored the characteristics of 179 DYW subgroup *PPR* genes, including intron-exon organization, chromosomal location and phylogenetic relationships. The DYW subgroup *PPR* genes were divided into three discrete groups (Cluster I to III). An analysis of a complete set of the Cluster I PPR genes/proteins was performed, including classification, chromosomal location, orthologous relationships, duplication analysis and tissue-specific expression patterns. *GmPPR4* was further investigated owing to significant up-regulation by drought and salt stresses. Our results showed that overexpression of *GmPPR4* improved tolerance to drought stress in soybean. Our study provided an insight into the foundation of the *GmPPR4* gene in abiotic stress responses.

## 2. Result

### 2.1. Identification of DYW Subgroup PPR Genes in the Soybean Genome

In our study, 205 proteins were identified in the soybean genome (*Glycine max* Wm82.a2.v1). The candidate members were examined for the required DWY domain, which was unique to DYW subgroup PPR proteins. SMART and Pfam databases were used to verify the presence of the conserved domain in all 205 DYW subgroup PPR proteins, and the results showed that a total of 197 members were identified. Except for the presence of conserved PPR domains, there are great differences in the size and physicochemical properties of the DYW subgroup *PPR* genes (Table S1). The statistical results showed that the amino acid sequence length of the 179 DYW PPR proteins varied from the lowest of 118 to the highest of 1431, the isoelectric point (p*I*) varied from 5.28 to 9.36, and the molecular weight (MW) ranged from 13.7 kDa to 160.3 kDa. The detailed information of the 179 DYW subgroup *PPR* gene sequences is provided in Table S1. For convenience, the 179 DYW subgroup *PPR* genes were named from *GmPPR1* to *GmPPR179* according to the order of their chromosomal locations [23,24].

**Figure 1:**
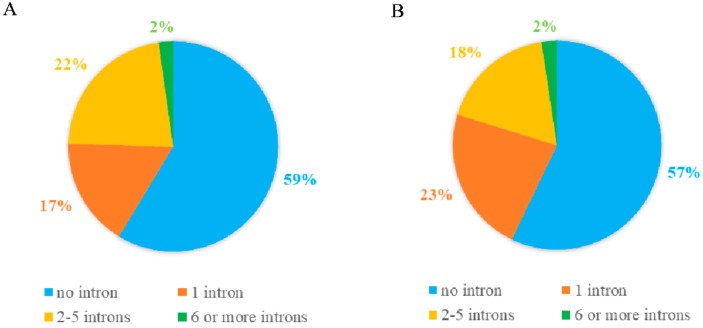
Relative proportions of intron-containing *PPR* genes in soybean. (**A**) Number of introns in the 179 DYW subgroup *PPR genes*. (**B**) Number of introns in the 84 Cluster I *PPR genes*.

A considerable amount of research showed that the vast majority of *PPR* genes were found to be lacking or containing several introns in various plant species [5,6,7]. The intron numbers present within the ORF of each of DYW subgroup *PPR* genes in soybean were decided for analyzing their exon-intron organization. As showed in Figure 1, 59% (105/179) of DYW subgroup *PPR* genes were predicted to lack introns, 17% (30/179) with one intron, 22% (40/179) with two to five introns, and 2% (4/179) with six or more introns.

### 2.2. Chromosomal Distribution, Phylogenetic Analysis and Multiple Sequence Alignment

**Figure 2:**
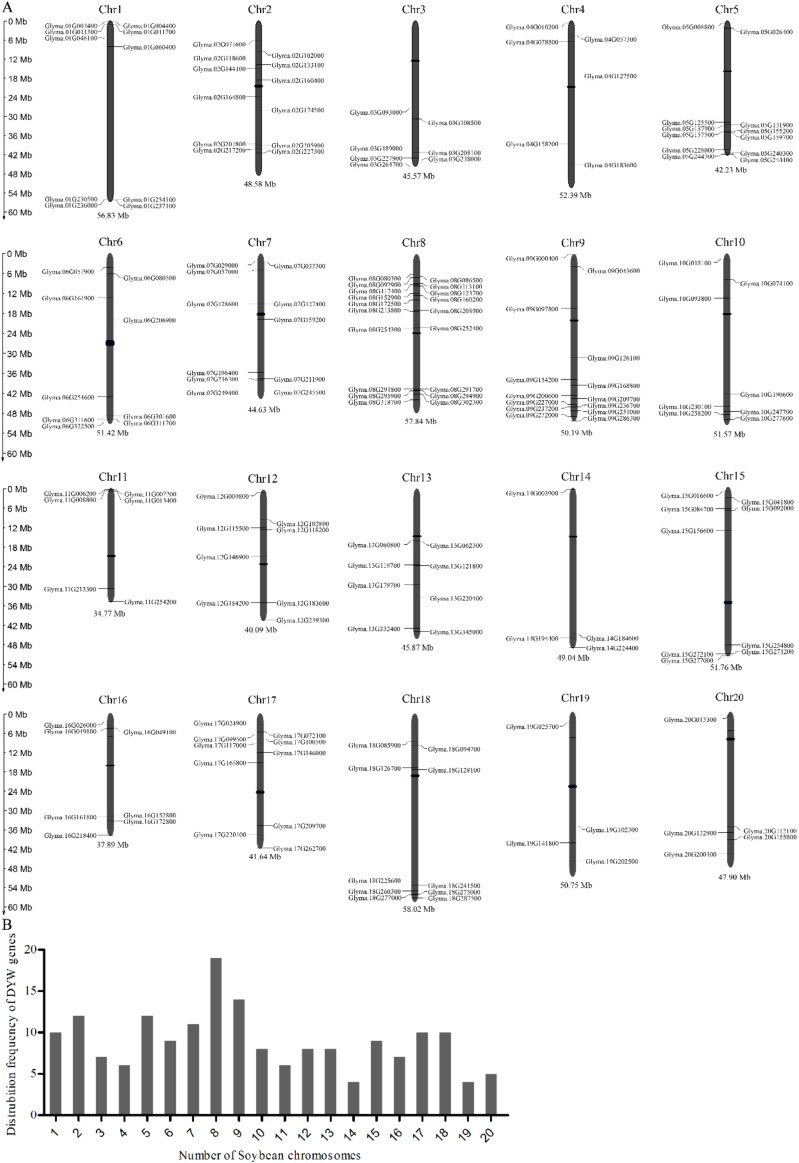
Chromosomal distribution of the 179 DYW subgroup *PPR* genes in soybean. (**A**) The physical location of each member. (**B**) *PPR* genes distribution on 20 soybean chromosomes.

Our study showed 179 DYW subgroup *PPR* genes were distributed widely and unevenly in all the 20 soybean chromosomes (Figure 2). Chromosome 8 of soybean contained the highest number of DYW subgroup *PPR* genes, while fewer numbers of DYW subgroup *PPR* genes were located on chromosome 14 and 19. The detailed position of each DYW subgroup of *PPR* genes on the soybean chromosomes was obtained from Phytozome (Table S1).

**Figure 3:**
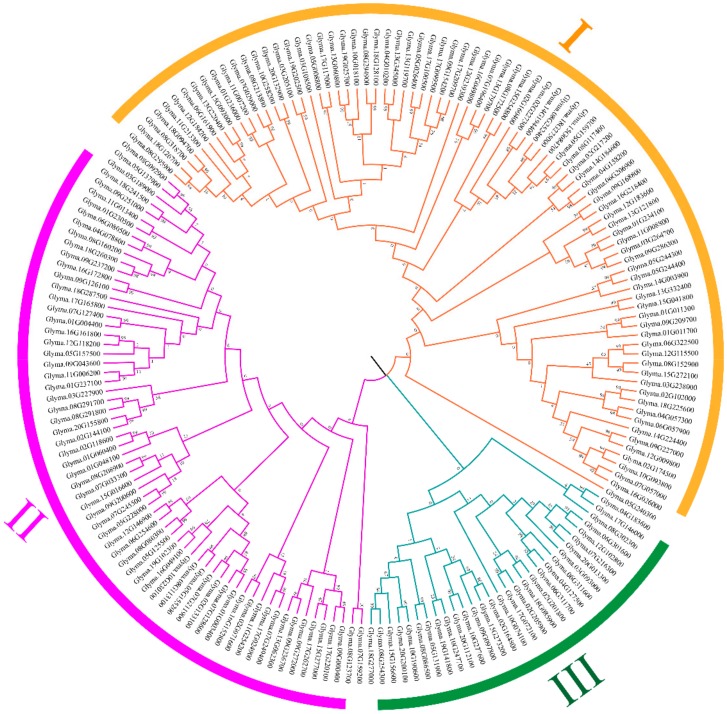
Phylogenetic tree of DYW subgroup *PPR* genes. The complete amino acid sequences of the 179 DYW subgroup PPR proteins were aligned by ClustalW and Maximum-likelihood with MEGA7. Three discrete groups (Cluster I to III) were highlighted in different colors.

To analyze the evolutionary relationship among DWY subgroup *PPR* genes, the full-length amino acid sequences of the 179 DYW subgroup PPR proteins were aligned and then the phylogenetic tree was conducted using the Maximum likelihood method by MEGA7.0 software (Figure 3). The phylogenetic analysis categorized the DYW subgroup *PPR* genes into three distinct clusters (I to III) comprising of 84, 65 and 30 genes, respectively. In order to isolate drought-inducible DYW subgroup *PPR* genes in soybean, we analyzed the expression patterns of the DYW subgroup *PPR* genes in soybean transcriptome database. We found that three DWY subgroup *PPR* genes were highly up-regulated by drought and salt stresses, including *GmPPR4* (Glyma.01G011700), *GmPPR18* (Glyma.02G174500), and *GmPPR111* (Glyma.11G008800). Interestingly, they all clustered into the Cluster I, implying that Cluster I *PPR* genes may be involved in the abiotic stress of soybean, therefore, we further analyzed the genetic structure and motif of Cluster I *PPR* genes.

**Figure 4:**
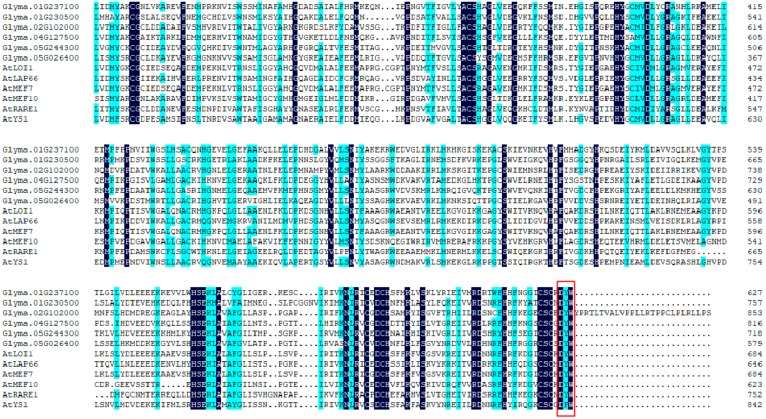
Multiple sequence alignment of 12 DYW subgroup PPR proteins from *A. thaliana* and soybean by DNAMAN. Black or blue shadings represent the 100% or >75% similarity of amino acids. Red rectangle marks highly core short signatures.

To identify conservation of PPR proteins in dicot plant species, six reported DYW proteins of *A. thaliana* and six randomly selected proteins of soybean were used to conduct the multiple sequence alignment, and the C-terminal region is displayed in Figure 4. Results showed that PPR proteins were relatively conserved across *A. thaliana* and soybean, however, only the significant short arrays (no more than four amino acid residues) were absolutely conservative, and 11 members harbored the conservative DYW sequence in C-terminal region that have previously been described as characteristic features of DYW subgroup PPR proteins. In addition, a phylogenic tree was conducted to reveal the relations of 12 selected proteins (Figure S1).

### 2.3. Gene Structure and Motif Composition of Soybean Cluster I PPR Genes

**Figure 5:**
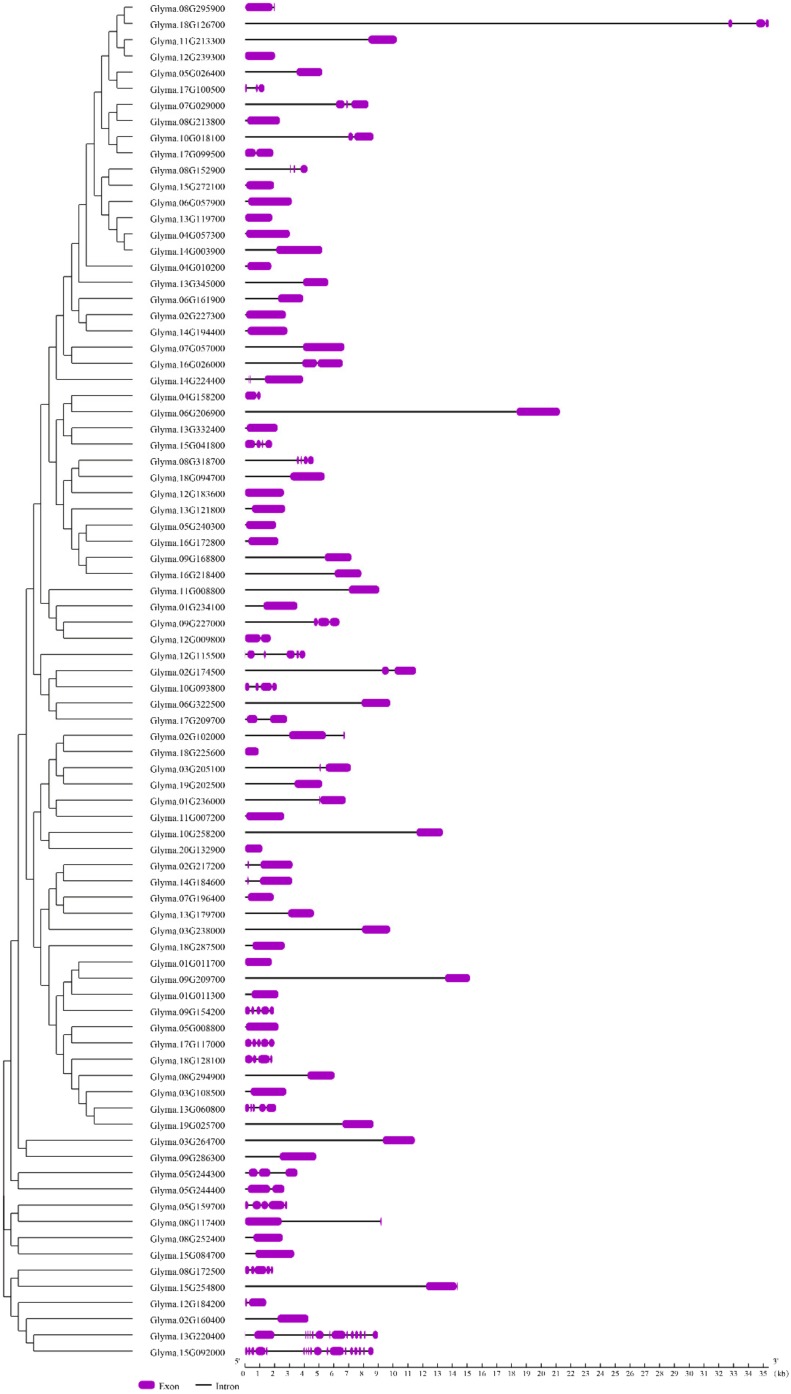
Phylogenetic relationship and structural analysis of the 84 Cluster I *PPR* genes. The phylogenetic tree was constructed via MEGA7.0 software; the different classes of *PPR* genes make up separate clades. The schematic diagram was carried out to represent the gene structure. Introns and exons were indicated by black lines and purple boxes, respectively. The lengths of introns and exons of each gene were displayed proportionally.

The exon-intron organization of the 84 Cluster I *PPR* genes was conducted to provide valuable information for the structure diversity evolution of the PPR family in soybean. As showed in Figure 5 and Table S1, they almost contained very few introns, which was similar to the previous results (Figure 1). However, the result showed that several genes contained more than 10 introns. Fourteen and 17 introns were found in Glyma.13G220400 and Glyma.15G092000, respectively. Many paralogs exhibited the same numbers and sizes of introns such as gene clusters of Glyma.07G057000, Glyma.16G026000, Glyma.02G217200, and Glyma.14G184600; However, only a few of them showed intron gain/loss phenomenon such as Glyma.03G205100 and Glyma.19G202500. On the whole, the intron distribution and the intron phase are in accordance with the alignment cluster of the Cluster I *PPR* genes.

**Figure 6:**
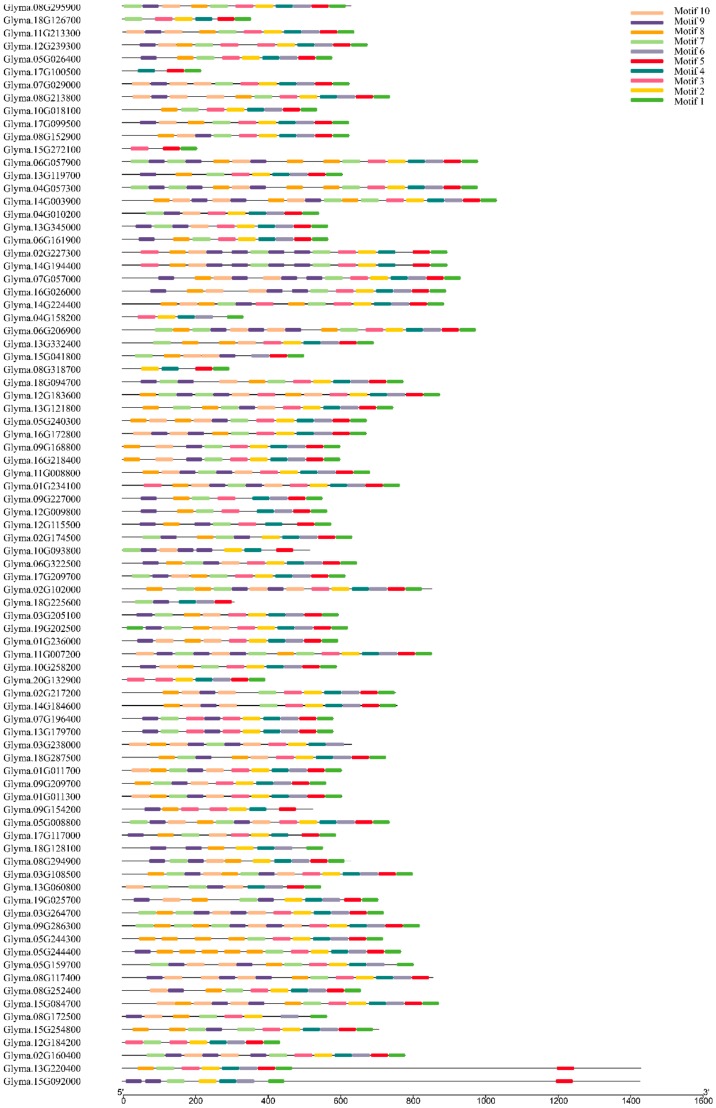
Putative motifs of each Cluster I PPR gene by MEME program and TBtools software. Ten putative motifs were indicated in colored boxes. The length of protein can be estimated using the scale at the bottom.

**Figure 7:**
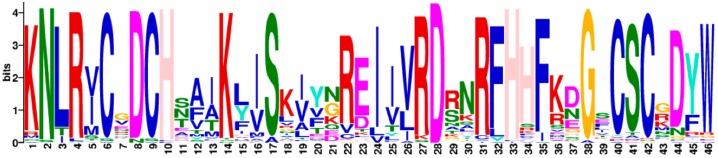
Sequence logo of global multiple alignment of 179 DYW subgroup PPR proteins by MEME program.

Also, the Multiple EM for Motif Elicitation (MEME) program was used to search the conserved motifs which were shared with the Cluster I proteins (Figure 6). A total of 10 distinct conserved motifs named 1 to 10, were found. The motifs were defined based on sequence conservation without knowing its structure or function. Motif 1, the most typical DYW domain, comprised of 46 amino acids; the sequence (KNLRVCGDCHSAIKLISKIYNREIIVRDRNRFHHFKBGSCSCGDYW) contains a highly conserved C-terminal region (DYW) (Figure 7). However, this conservative motif is not included in the five proteins, including GmPPR28 (Glyma.03G238000), GmPPR72 (Glyma.08G117400), GmPPR91 (Glyma.09G154200), PPR103 (Glyma.10G093800), and GmPPR165 (Glyma.18G225600), which may be due to the fact that some PPR proteins are truncated at the C-terminus, lacking part, most of or the entire DYW domain. Proteins with higher homology often have the same conserved domain composition, which corresponds to the results of the phylogenetic tree.

### 2.4. Duplication and Divergence Rate of Soybean Cluster I PPR Genes

**Figure 8:**
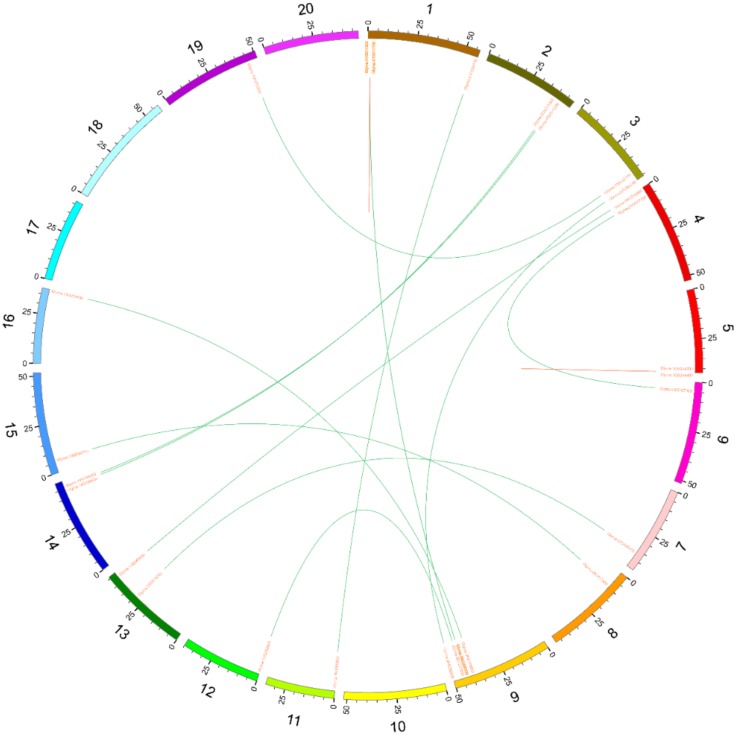
Distribution of segmentally duplicated Cluster I *PPR* genes on soybean chromosomes. Green lines indicate duplicated *PPR* gene pairs, and bold Photozome Locus indicates the gene appears twice.

Whole genome tandem and segmental duplications play a vital role in multiple copies of genes in a gene family and their subsequent evolution. Among *SiPPR* genes, 190 (95pairs; ~39.01%) were segmentally duplicated [25]. Our research reached a similar conclusion that this proportion is far higher than of genes generally. Among Cluster I *PPR* genes, 30 (15 pairs; ~35.7%) were segmentally duplicated (connected by lines in Figure 8). Among 15 paralog pairs, 13 segmental duplication gene pairs were involved in two chromosomes; only two segmental duplications were intra-chromosomal. In particular, three genes have appeared twice, including *GmPPR3* (Glyma.01G011300), *GmPPR4* (Glyma.01G011700) and *GmPPR94* (Glyma.09G209700). The results indicated that gene segmental duplications might mainly contribute to the expansion of the PPR gene family in soybean.

The ratio of non-synonymous (Ka) versus synonymous (Ks) substitution rates (Ka/Ks) was estimated for 15 segmental duplicated gene pairs to evaluate the role of Darwinian positive selection in duplication and divergence of the Cluster I *PPR* genes (Table S2). The result showed that the Ka/Ks for segmental duplications gene-pairs ranged from 0,14 to 1.03 with an average of 0.61. On the whole, it suggested that the duplication Cluster I *PPR* genes were under purifying selection pressures since their Ka/Ks rations were estimated as <1 except one gene pair with a rate (Ka/Ks) of 1.03.

### 2.5. Tissue-Specific Expression Pattern of DYW Subgroup PPR Genes

**Figure 9:**
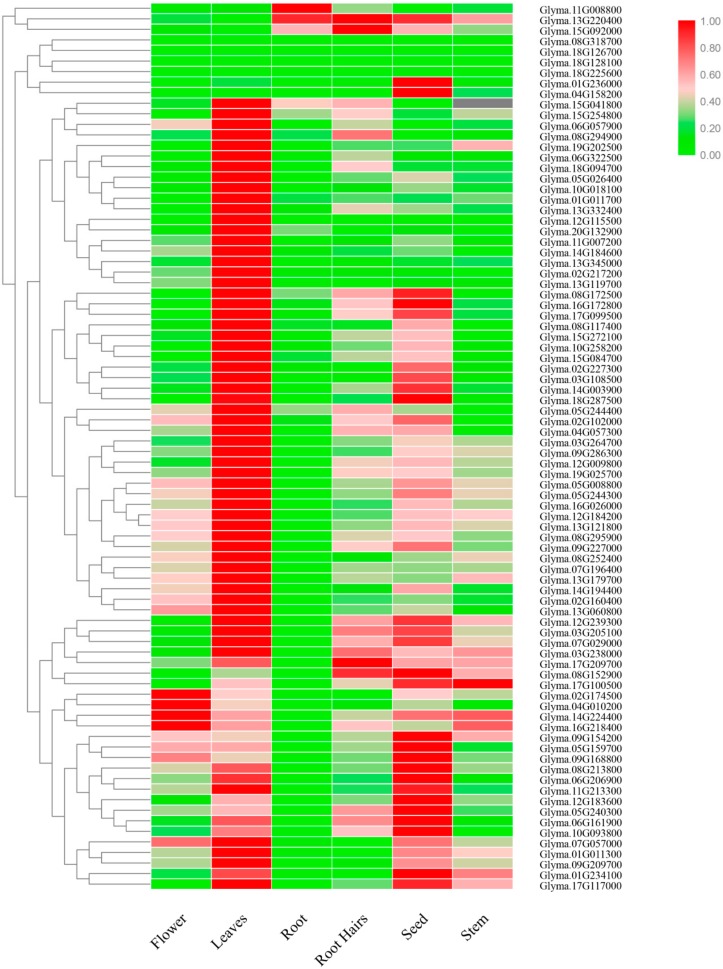
Heat map of expression profiles (in log10-based FPKM) of all DYW subgroup *PPR* genes from six soybean tissues (flower, leaves, root, root hairs, seed, and stem). The expression abundance of each transcript is represented by the color bar: Red, higher expression; and green, lower expression.

Different members may exhibit significant diversity in expression abundance among different tissues to adapt to different physiological process. To gain insight into the gene expression patterns within the organism in soybean growth and development, we investigated the relative transcript abundance of the 84 DYW subgroup *PPR* genes in six soybean tissues using publicly available RNA-seq data from Phytozome database, including flower, leaves, root, root hairs, seed, and stem, revealing that most DYW subgroup *PPR* genes had a similar pattern of expression in the same tissue. Out of 84 DYW subgroup *PPR* genes, most of them expressed in almost any tissues, while a total of four genes had no expression abundances to be discovered in any tissues, including Glyma.08G318700, Glyma.18G126700, Glyma.18G128100, and Glyma.18G225600. We found that most *PPR* genes showed preferential accumulation in leaves compared to other tissues, which were likely to be responsible for their subcellular localization, and most reported PPR proteins were located in mitochondria or chloroplast. A relatively high expression level was revealed in seeds, suggesting that they have potential function in seed development, which has been reported to be involved in endosperm development [14]. As shown in Figure 9, Glyma.11G008800, Glyma.13G220400 and Glyma.15G192000 showed extremely high expression in roots and root hairs, indicating that the DYW subgroup *PPR* genes may play specific roles in responding to drought stress.

### 2.6. Cis-Elements Analysis

**Figure 10:**
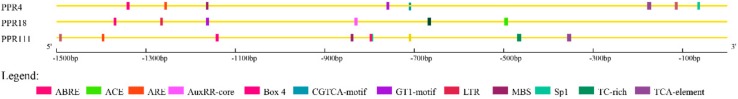
Putative *cis*-element in a 1.5 kb 5′ flanking region upstream from the start codon. Different *cis*-elements were indicated by colored symbols and placed in relative positions on the promoter. The ABA-responsive element (ABRE), light-responsive element (ACE), anaerobic induction element (ARE), auxin responsive element (AuxRR-core), light-responsive element (Box4), MeJA-responsive (CGTCA-motif), light-responsive element (GT1-motif), low temperature responsive element (LTR), MYB-binding site (MBS), light-responsive element (Sp1), salicylic acid responsive element (TCA), and defense and stress responsive element (TC-rich repeat) were analyzed.

By examining the expression levels of DYW subgroup *PPR* genes according to soybean drought- and salt-induced transcriptome database, we found that three genes were highly up-regulated to drought and salt stresses. *Cis*-elements presented in the upstream region play major roles in regulating the gene expression at the transcriptional level. To further understand the mechanism of the three genes, a set of 12 important *cis*-elements were identified in their 1.5 Kb 5′ flanking region upstream from the start codons. Various *cis*-elements including ABA-responsive element (ABRE) and MYB-binding site (MBS) were shown in Figure 10, which suggested that the three candidate genes might be involved in responses abiotic stresses.

### 2.7. Several Candidates Are Involved in Abiotic Stresses

**Figure 11:**
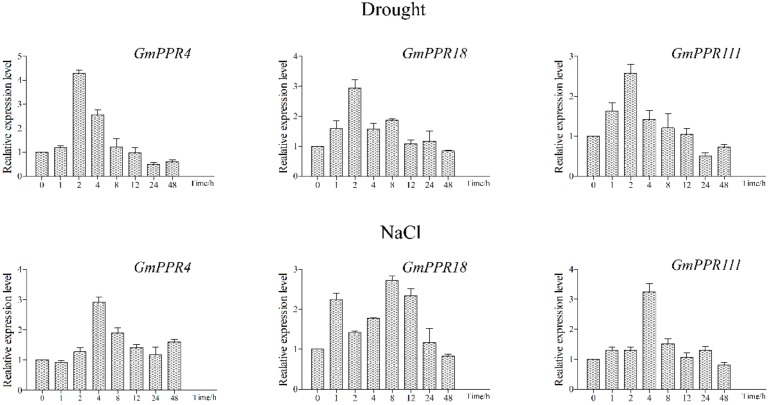
Expression patterns of three selected *PPR* genes under salt and drought treatments by qRT-PCR. *GmPPR4* (Glyma.01G011700), *GmPPR18* (Glyma.02G174500), *GmPPR111* (Glyma.11G008800), respectively. The *actin* gene was used as an internal control. The data are shown as the means ± SD obtained from three biological replicates. ANOVA test demonstrated that there were significant differences (* *p* < 0.05, ** *p* < 0.01).

To comprehensively understand the physiological functions of DYW subgroup *PPR* genes, we initially examined the expression patterns of three genes in response to salt and drought stresses by qRT-PCR (Figure 11). Under drought treatment, these three genes exhibited similar expression patterns, reaching a peak at 2 h (~ 4.5-fold, 3-fold and 2.5-fold, respectively), indicating that these genes have a similar biological function in the same environment. Similarly, the expression levels of the three genes were enhanced by salt; the expression peaks of *GmPPR4/18/111* occurred at 4, 8 and 4 h, respectively, which are equivalent to 3-fold, 2.8-fold and 3.2-fold increases, respectively.

### 2.8. GmPPR4 Improved Drought Tolerance in Transgenic Soybean Hairy Roots

**Figure 12:**
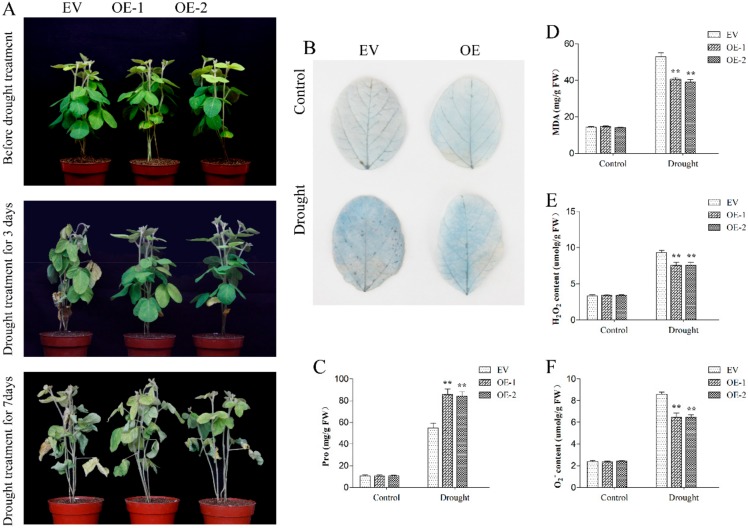
Drought stress analysis of EV-control transgenic plants and *GmPPR4*-OE transgenic plants. (**A**) Phenotypes of *GmPPR4*-OE and EV-control transgenic soybean plants subjected to drought stress without water for 3 days and 7 days. (**B**) Trypan blue staining of soybean plant leaves placed on filter paper for the induction of rapid drought for 3 h, the dead cells can be strained, but living cells cannot. (**C**) The proline content, (**D**) malondialdehyde (MDA) content, (**E**) H_2_O_2_ content, and (**F**) O_2_^−^ content of *GmPPR4*-OE and EV-control transgenic soybean plants under normal and drought conditions. The data are shown as the means ± SD obtained from three biological replicates. ANOVA test demonstrated that there were significant differences (* *p* < 0.05, ** *p* < 0.01).

Among the three genes, *GmPPR4* (Glyma.01G011300) clearly responded to drought and salt stresses. For this reason, *GmPPR4* was selected for further investigation. To examine the function of *GmPPR4* in vivo, transgenic soybean plants which overexpressed *GmPPR4* (*GmPPR4*-OE) were generated into soybean hairy roots [26]. qRT-PCR analysis showed that *GmPPR4* accumulated in the *GmPPR4*-OE plants. (Figure S2), and about 80% of the roots of transgenic soybean plants were positive. To examine whether *GmPPR4* plays a role in the drought stress tolerance, we compared drought tolerance of *GmPPR4*-OE and WT plants at the vegetable stage; the hairy roots of seedlings which grew in soil were withheld from water for 2 weeks. No significant differences were observed for transgenic plants under normal growth conditions, compared to empty vector (EV)-transformed control hairy roots; drought treatment caused obvious differences in the growth of the EV-control and *GmPPR4*-OE plants; compared with the *GmPPR4*-OE, the EV-control plants showed wilted leaf under drought for 3 days, and seriously dehydrated leaves were observed after 7 days, whereas the *GmPPR4*-OE seedlings showed delayed and less leaf rolling during the drought stress process. (Figure 12A).

To explore the potential physiological mechanism responsible for the improved drought tolerance of the *GmPPR4*-OE seedlings, we compared some stress-related physiological changes in the EV-control and *GmPPR4*-OE plants under both normal growth and drought conditions. We found that proline accumulation in the transgenic plants was much more evident than in EV-control plants (Figure 12C), while the MDA content was decreased due to drought stress (Figure 12D). Furthermore, we also measured the level of H_2_O_2_ and O_2_^-^ in roots; we found that the drought-treated EV-control roots accumulated much more H_2_O_2_ and O_2_^-^ than the *GmPPR4*-OE roots (Figure 12E,F).

In addition, we used Trypan blue solution to detect cell activity in EV-control and *GmPPR4*-OE leaves. As shown in Figure 12B, no plant leaves differed under drought stress conditions, however, the color depth of the *GmPPR4*-OE leaves was lower than EV-control leaves under drought treatment, which indicated that the cell membrane integrity and stability in the leaves of the EV-control plants was better than that in the leaves of the *GmPPR4*-OE plants.

NaCl treatment was carried out. However, no obvious differences were observed in EV-control and *GmPPR4*-OE plants under NaCl treatment.

### 2.9. GmPPR4-OE Plants Exhibited Increased Transcripts of Some Drought-Inducible Genes

**Figure 13:**
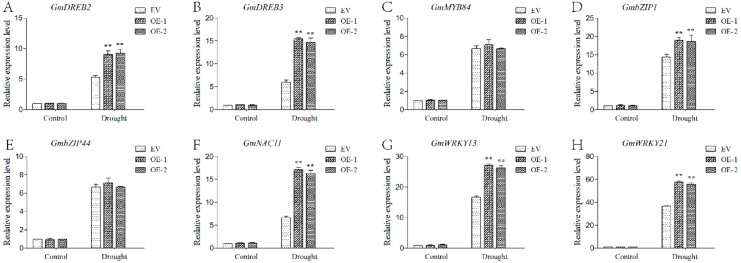
GmPPR4-OE plants exhibited increased transcripts of some drought-inducible genes. The two-week-old soybean seedlings were placed on filter paper for 3 h. qRT-PCR analysis for drought-inducible genes (**A**–**H**). The actin gene was used as an internal control. The data were shown as the means ± SD obtained from three biological replicates. ANOVA test demonstrated that there were significant differences (* *p* < 0.05, ** *p* < 0.01).

A previous study indicated that several genes play an important role in drought stress [27,28], including *DREB2* [29], *DREB3* [30], *MYB84* [31], *bZIP1* [32], *bZIP44* [33], *NAC11* [34], *WRKY13* [35], and *WRKY21* [35,36]. We compared the transcripts of several drought-inducible maker genes between *GmPPR4*-OE and EV-control plants under normal and drought conditions, and we found that drought induced more transcripts of all genes. However, expression differences between *GmPPR4*-OE and EV-control plants under drought treatment occurred in six genes, including *DREB2*, *DREB3*, *bZIP1*, *NAC11*, *WRKY13*, and *WRKY21*. There was no clear difference in *bZIP44* and *MYB84* expression with drought treatment between *GmPPR4*-OE and EV-control plants (Figure 13).

## 3. Discussion

Soybean is one of the most widely cultivated crops, with a total production of more than 260 million tons in 2010 (FAO data) [37]. Greenhouse and field studies showed that drought stress seriously affects plant development and led to significant reduction in crop yield [38]. Thus, when identifying ideal candidate genes, increasing drought stress resistance is essential for improving soybean yield.

*PPR* genes comprise a large family, which is ubiquitous to all terrestrial plants. In particular, previous reports indicated that a total of 4000 *PPR* family genes were identified in the spikemoss (S. *moellendorffii*) genome [3]. It is estimated that there were more than 1000 *PPR* genes in soybean. The duplication event was likely to be responsible for the large PPR family size, which has been experimentally verified in many species. Genome-wide identification of *PPR* family genes has been widely carried out in many species that have been sequenced [5,6,7]. In the present study, we identified 179 DYW subgroup *PPR* genes in the soybean genome (*Glycine max* Wm82.a2.v1). Consistent with previous studies, gene structure analysis revealed that most members of 179 DWY subgroup *PPR* genes were intron-less. Approximately 80% and 65% of *PPR* genes were free of intron in *A. thaliana* and rice, respectively [5,6]. The intron-less nature of the great majority of *PPR* genes may be the result of selection pressures during evolution. However, there are several *PPR* genes that tend to evolve diverse exon-intron organizations with more than 10 introns; we propose that they have a higher probability of evolving new specialized functions and adjusting to their living environment. The intron-less nature may demonstrate a duplication event of *PPR* genes [39]; we presumed that the nature also allows alternative splicing and alteration of splicing pattern in plants. In particular, many coding sequences of PPR proteins with extremely high similarity on the genome were divided only by frameshifts, for example, Glyma.13G332400 and Glyma.15G041800. In our study, *GmPPR3* (Glyma.01G011300), *GmPPR4* (Glyma.01G011700) and *GmPPR94* (Glyma.09G209700) shared more than 95% identity in their coding sequence. In particular, many *PPR* genes have extremely close proximity on the genome, which contributed to the extensive gene-level synteny shared between them [24].

Previous studies have shown that PPR proteins were involved in RNA editing, plant growth and development and abiotic stress. Several PPR proteins have been implicated with trans-splicing of *nad* intron in *A. thaliana*, these include: OTP43 [40], SLO4 [41], PPR19 [42], BIR6 [43], MTL1 [44], ABO5 [21], OTP439 [45], and SG3 [46]. Lots of PPR proteins have a similar function in the development of seed or endosperm, such as EMP5 [47], PPR2263 [48], PPR8522 [49], OGR1 [50], AHG11 [17], and OTP43 [40]. In addition, in rice or *A. thaliana*, six PPR proteins: cisc(t) [20], ABO5 [21], ECB2 [51], PPR40 [22], SVR7 [52], and YS1 [53], were reported to be involved in abiotic stress responses, including ABA, drought, light and cold. In this study, a higher level of H_2_O_2_ and O_2_^-^ were found in *GmPPPR4*-OE plants compared with EV-control plants under drought stress. In a previous study, the *abo5* mutant accumulated higher H_2_O_2_ content in roots than wild type [21], and the *ahg11* mutants exhibited higher transcript levels of oxidative stress-responsive genes [17]. The above results suggested that *PPR* genes may play roles in drought stress by regulating the level of H_2_O_2_.

## 4. Methods

### 4.1. Identification of DYW Subgroup PPR Genes in Soybean

DYW subgroup PPR proteins sequences of soybean were downloaded from Phytozome [54]. Predicted proteins from the soybean genome (*Glycine max* Wm82.a2.v1) were scanned using HMMER v3 [55] using the Hidden Markov Model (HMM) corresponding to the Pfam of PPR family (PF14432) [56]. The proteins obtained using the raw DYW HMM, a high-quality protein set (E-value < 1 × 10^−20^), were aligned and used to construct a soybean-specific DYW HMM profile using hmmbuild from the HMMER v3 suite. This new soybean-specific HMM was used to search for all members in all soybean proteins; in addition, all obtained proteins with an E-value lower than 0.01 were selected. The highly matched sequences were reorganized and merged to remove the redundancy. Then, all protein sequences of putative DYW subgroup *PPR* genes were submitted to the SMART database [57] and Pfam database to confirm the existence of the DYW conserved domain. The sequences lacking a DYW domain were refused in this study. Furthermore, molecular weights and isoelectric points identified that the DYW subgroup PPR proteins were obtained by using tools from EXPASY website [58].

### 4.2. Chromosomal Location and Phylogenetic Analysis

Positional information of DYW subgroup *PPR* genes on chromosomes of soybean was obtained from the Phytozome database. All DYW subgroup *PPR* genes were mapped to 20 chromosomes in soybean.

Multiple sequence alignment of proteins from soybean was performed via ClustalW with a default parameter. A phylogenetic tree was constructed by using the maximum likelihood with MEGA7 software, with the following parameters: Poisson model, pairwise deletion and 1000 bootstrap replications.

### 4.3. Gene Structure Analysis and the Sequence of PPR Motif Analysis

The gene structures of *PPR* were illustrated using the online program Gene Structure Display Server [59] by comparing predicted coding sequences with their corresponding genomic DNA sequences.

For sequence analysis of PPR motifs, the MEME online program [60] was used to identify conserved motifs. The scatter diagram was used to display the distribution of PPR motifs at the opposite positions in the PPR proteins by using the TBtools software v0.665 (Guangzhou, China) [61].

### 4.4. Gene Duplication

All Cluster I PPR proteins were searched for using the BLASTp search (E-value >1e^−10^) with more than 75% sequence similarity being considered to be a pair of tandem repeat genes. Then, the resulting file and GFF3 files of soybean genome (*Glycine max* Wm82.a2.v1) were used to analyze the gene duplication by software MCScanX and visualization using CIRCOS [62].

### 4.5. Tissue-Specific Expression Patterns of DWY subgroup PPR genes

Transcription databases were obtained from Phytozome database to investigate the tissue expression patterns of the soybean DYW subgroup PPR family; TBtools software was used to conduct a visual hierarchical clustering of the 84 DYW subgroup *PPR* genes under normal conditions. The transcript data are available in Table S3.

### 4.6. Promoter Sequence Analysis for Potential cis-Elements

For *cis*-elements analysis, 1.5 kb 5′ upstream region sequences were extracted from the Phytozome database. Then, the potential *cis*-elements of promotors for each gene were analyzed using PlantCARE database [63].

### 4.7. Plant Materials and Treatments

Soybean cultivar Zhonghuang 39 was used to analyze the gene expression pattern. The leaves of 15-day soybean seedlings were collected for RNA extraction and used to further qRT-PCR analysis. For drought stress, the soybean seedlings were placed on filter paper, for NaCl stress, the soybean seedlings were subjected to a 200 mM NaCl solution; samples were collected at 0, 1, 2, 4, 8, 12, 24, and 48 h after treatments. All treated leaf samples were frozen immediately in liquid and then stored at −80 °C for subsequent analysis.

### 4.8. RNA Extraction and qRT-PCR

Total RNA was extracted from plant samples using the Trizol method as the manufacturer’s protocol (TIANGEN, Beijing, China), which was reverse transcribed into cDNA using a PrimeScriptTM RT Reagent Kit (TaKaRa, Shiga, Jappa). All primers used in the study are shown in Table S4 [64].

### 4.9. Agrobacterium Rhizogenes-Mediated Transformation of Soybean Hairy Roots

Soybean Williams 82, a typical variety, was used to generate *GmPPR4*-OE soybean hairy roots. The coding sequences of *GmPPR4* was ligated into plant transformation vector pCAMBIA3301 with the CaMV 35S promoter. The constructs were transferred into A. rhizogenes strain K599, and *Agrobacteriumrhizo* strain K599 harboring EV-control and *GmPPR4*-OE were injected at the cotyledonary node, as previously described [65]. The injected plants were transferred to the greenhouse with high humidity until hairy roots were generated at the infection site and had grown to about 5 cm long. The original main roots were cut off from 0.5 cm below the infection site. Seedlings were transplanted into nutritious soil and cultured normally in the greenhouse for a week (25 °C 16 h light/8 h dark photoperiod).

### 4.10. Drought and Salt Stress Assays

For drought treatment, one-week-old seedlings were subjected to dehydration for 15 days. After drought treatment, we re-watered soybean plants for 3 days. At the same time, we carried out salt treatment with 200 mM NaCl solution for 3 days [66].

### 4.11. Measurement of Proline Content, MDA Content, H_2_O_2_ Content and O_2_^-^ Content

The content of proline, MDA, H_2_O_2_ and O_2_^-^ were measured with the corresponding assay kit (Cominbio, Suzhou, China) based on the manufacturer’s protocols; all measurements were from four biological replicates.

### 4.12. Trypan Blue Staining

The leaves separated from the EV-control and *GmPPR4*-OE plants were placed on filter paper for the induction of rapid drought for 3 h, which were used to trypan stain, as previously described [23].

## 5. Conclusions

This study is the first time identifying the presence of 179 DYW subgroup *PPR* genes in the soybean genome (*Glycine max* Wm82.a2.v1) sequences, which were designated to *GmPPR1* through *GmPPR179* on the basis of their chromosomal location. We conducted a comprehensive and systematic analysis of the DYW subgroup PPR family. Based on the gene expression patterns under drought and salt stresses, we found that three *PPR* genes were highly up-regulated under salt and drought treatment, and *GmPPR4* was selected for validating its role in drought stress tolerance. Compared with the EV-control plants, *GmPPR4*-OE exhibited drought tolerant phenotypes. Our results showed that *GmPPR4* could improve tolerance to drought in soybean.

## Supplementary Materials

Supplementary materials can be found at https://www.mdpi.com/1422-0067/20/22/5667/s1. Table S1. DYW-subgroup *PPR* genes in soybean. Detailed genomic information including Phytozome locus, class present, number of introns within ORF, protein, genomic locus (chromosomal location), +/− stand, isoelectric point, and molecular weight (kDa) of the PPR proteins for each *PPR* gene. Table S2. The Ka/Ks ratios for segmentally duplicated the Cluster I PPR proteins. Table S3. Transcript data of 84 DYW subgroup *PPR* genes for heat map. Table S4. The sequences of primers used in the study. Figure S1. The phylogenic tree of 12 selected proteins. Figure S2. qRT-PCR analysis of *GmPPR4* expression in *GmPPR4*-OE and EV-control transgenic hairy roots.

## Abbreviations

NCBI	National Center for Biotechnology Information
PPR	Pentatricopeptiderepeat
qRT-PCR	Quantitative real-time PCR
ABA	abscisic acid
MDA	malondialdehyde
Pro	proline
