# Supplementary material for: Genome-Wide Analysis of the DYW Subgroup PPR Gene Family and Identification of GmPPR4 Responses to Drought Stress
Source: Int J Mol Sci. 2019 Nov 12;20(22):5667. doi: 10.3390/ijms20225667 (PMC6888332; doi:10.3390/ijms20225667)
Supplement: Supplementary file 1 [file ijms-20-05667-s001.zip › ijms-610409-supplementary-xml/Figure S1,S2 and Table S4.pdf]

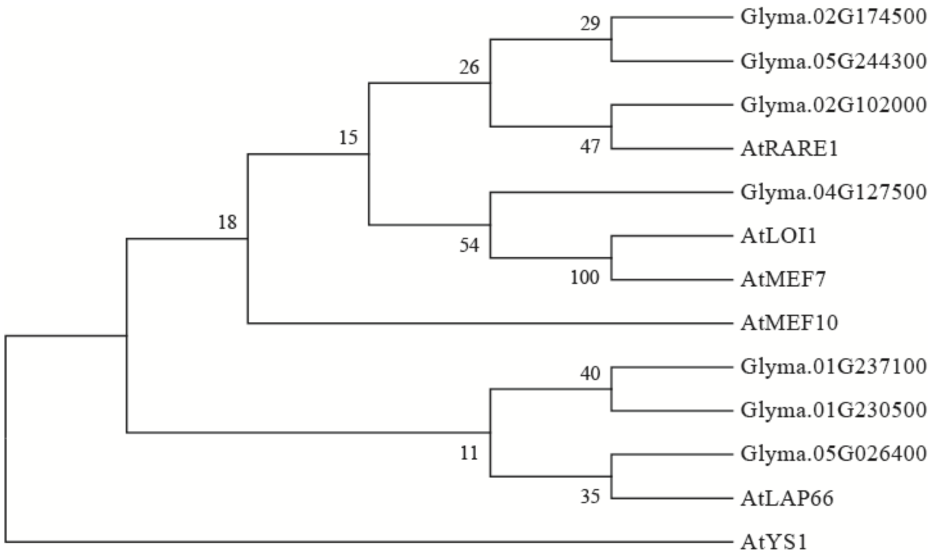

Figure S1. The phylogenic tree of 12 selected proteins.

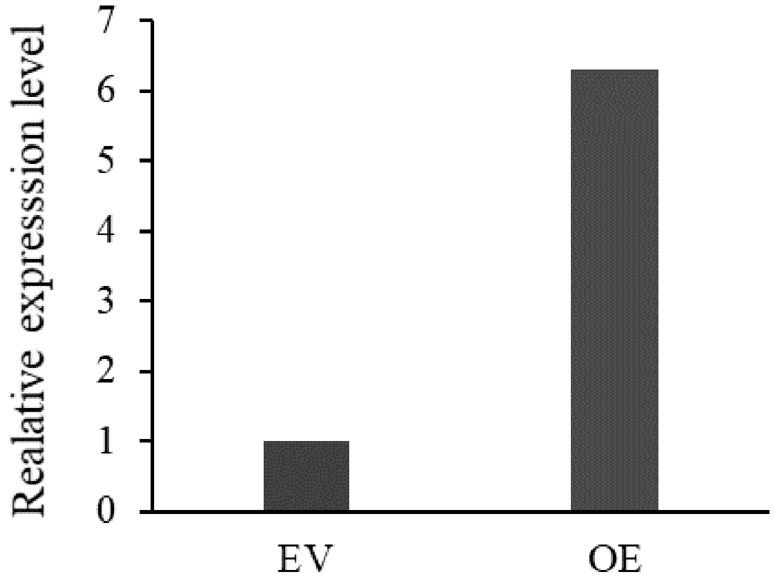

Figure S2. qRT-PCR analysis of *GmPPR4* expression in *GmPPR4*-OE and EV-control transgenic hairy roots.

Table S4. The sequences of primers used in the study.

**For gene cloning**	
PPR4F	ATGGGCGGTATGCTATTT
PPR4R	AAAATACTACCCCTACAA
**For vector construction**	
PPR4-OEF	GGACTCTTGACCATGATGAGTGGGACATCT
PPR4-OER	ATTCGAGCTGGTCACCCCAATAATCTTTACA
**For quantitative RT-PCR**	
RT-PPR4F	TTCTTGGGGACATGAGCAGG
RT-PPR4R	TCAAGGCTCCCACACTTGAC
RT-PPR18F	ACGAGTGCCTGAGGAAAAGG
RT-PPR18R	GTCCACGACACCACATTCCT
RT-PPR111F	CTTGGCTTGGAATCCGAGGT
RT-PPR111R	GCAGCTTCAAACCTTGCACA
GmactinF	ACATTGTTCTTAGTGGTGGCT
GmactinR	CTGTTGGAAGGTGCTGAG
DREB2F	AGCGAAAGCAGCAGCACC
DREB2R	GTTAAGGCGAGCGGAAGG
DREB3F	CCGCAGTGGGAAGGTGATT
DREB3R	CAGTAGCAGCCACCTGAGAA
MYB84F	GGGGAAACAGGTGGTCAA
MYB84R	TCTAGGCATCCAGAAACG
bZIP1F	GAGGTTTGGAGGGACTTG
bZIP1R	TTGAGGATTTGATGAGCC
bzip44F	TCGGATGCGAAAGCGTAA
bzip44R	TGCGTGGTGATGTCTATGGTG
NAC11F	TCCCTCTTGTTCTATGCT
NAC11R	GTTTCCCATTATTTGCCTA
WRKY13F	AGGACACGGATACAGCAA
WRKY13R	CATCTTCAGGGTAGGCAC
WRKY21F	GATAACCGTCACTCTGCC
WRKY21R	AGTTTCCTTTCTCCGATG
